# Inducible *Rbpms*-CreER^T2^ Mouse Line for Studying Gene Function in Retinal Ganglion Cell Physiology and Disease

**DOI:** 10.3390/cells12151951

**Published:** 2023-07-27

**Authors:** Luming Guo, Xiaoling Xie, Jing Wang, Haiyan Xiao, Shuchun Li, Mei Xu, Ebenezer Quainoo, Rithwik Koppaka, Jiaping Zhuo, Sylvia B. Smith, Lin Gan

**Affiliations:** 1 College of Life Sciences, Zhejiang University, Hangzhou 310058, China; 2 Department of Neuroscience and Regenerative Medicine, Medical College of Georgia, Augusta University, Augusta, GA 30912, USA; 3 Department of Cellular Biology and Anatomy, Medical College of Georgia, Augusta University, Augusta, GA 30912, USA; 4 James and Jean Culver Vision Discovery Institute, Medical College of Georgia, Augusta University, Augusta, GA 30912, USA

**Keywords:** Cre recombinase, glaucoma, optic neuropathy, RGC and axonal degeneration, RGC development

## Abstract

Retinal ganglion cells (RGCs) are the sole output neurons conveying visual stimuli from the retina to the brain, and dysfunction or loss of RGCs is the primary determinant of visual loss in traumatic and degenerative ocular conditions. Currently, there is a lack of RGC-specific Cre mouse lines that serve as invaluable tools for manipulating genes in RGCs and studying the genetic basis of RGC diseases. The RNA-binding protein with multiple splicing (RBPMS) is identified as the specific marker of all RGCs. Here, we report the generation and characterization of a knock-in mouse line in which a P2A-CreER^T2^ coding sequence is fused in-frame to the C-terminus of endogenous RBPMS, allowing for the co-expression of RBPMS and CreER^T2^. The inducible *Rbpms*-CreER^T2^ mice exhibited a high recombination efficiency in activating the expression of the tdTomato reporter gene in nearly all adult RGCs as well as in differentiated RGCs starting at E13.5. Additionally, both heterozygous and homozygous *Rbpms*-CreER^T2^ knock-in mice showed no detectable defect in the retinal structure, visual function, and transcriptome. Together, these results demonstrated that the *Rbpms*-CreER^T2^ knock-in mouse can serve as a powerful and highly desired genetic tool for lineage tracing, genetic manipulation, retinal physiology study, and ocular disease modeling in RGCs.

## 1. Introduction

Retinal ganglion cells (RGCs), the sole output neurons of the retina, receive and integrate visual information from photoreceptors via bipolar and amacrine interneurons and project axons to the visual processing centers in the brain [1,2]. Most RGCs are localized in the ganglion cell layer (GCL) of the retina, and the remaining 2–3% of RGCs are displaced RGCs (dRGCs) residing in the inner nuclear layer (INL) at the outer border of the inner plexiform layer (IPL) [3,4]. RGCs consist of a heterogeneous population of cells with greater than 40 different subtypes that are identified based on the anatomical classification of cell morphology and dendritic stratification, input circuits, molecular profiles based on single-cell transcriptomics, and functional classification [5,6,7,8,9,10,11,12]. The diversity in RGCs and their complicated connections with upstream retinal interneurons form the elaborate circuitry to integrate vision signals precisely.

The dysfunction and degeneration of RGCs cause severe ocular diseases and lead to irreversible blindness, such as Leber Hereditary Optic Neuropathy (LHON) and Autosomal Dominant Optic Atrophy (ADOA) in inherited optic neuropathies as well as acquired optic neuropathies including glaucoma, demyelinating optic neuritis, and toxic optic neuropathy [13,14,15,16]. Up to now, studies of these clinical ocular diseases associate the vision impairment with prevalent mutations in mitochondrial DNA and *OPA1* in LHON and ADOA [13,17,18]. Genetic mutations in *MYOC*, *CYP1B1*, *PITX2*, *FOXC1,* and *OPTN* are linked to glaucoma [13,14,19], which is one of the most prevalent causes of visual impairment and blindness in the world [20] and is characterized by the degeneration of the optic nerve and RGCs. In addition, previous studies have shown that the transcription factors of the Class 4 POU-homeodomain proteins (POU4F1 and POU4F2), LIM-homeodomain member (ISL1), SRY-related HMG-box (SOX) proteins (SOX4, SOX11, and SOX12), and basic helix-loop-helix (bHLH) protein (ATOH7) participate in the development and specification of RGCs and that functional disruptions of these transcription factors lead to the loss of RGCs or axon deficits in mice [21,22,23,24,25,26,27,28,29,30]. With the application of single cell high throughput sequencing in RGCs, more and more gene features are identified among different subtypes of RGCs. However, the roles of genes and the genetic mechanisms underlying the development, maintenance, and pathogenesis of RGCs remain poorly understood. Genetic tools for manipulating gene expression specifically in RGCs are essential and of great benefit to the field. Currently, a few methods have been used for gene manipulation in RGCs, including viral infection [9,31,32,33] and gene-specific promotor-driven Cre mouse lines [34,35,36]. However, these existing tools have limitations, such as that transduction of viral vectors is limited by the delivery efficiency and toxicity and that the available Cre lines display limited Cre recombinase activities in selective RGCs but not in the entire population of RGCs [37,38,39,40,41], or they express Cre in other retinal cell types besides RGCs [42,43] or are not inducible for gene manipulation at a given time point [42,44]. Hence, there is a critical need for efficient inducible Cre mouse lines that can achieve highly effective recombination of floxed alleles specifically in RGCs in a temporally controlled manner.

RNA-binding protein with multiple splicing (RBPMS) contains a conserved RNA recognition motif and is involved in RNA splicing and the development of heart and smooth muscles [45,46]. Inactivation of *Rbpms* leads to perinatal lethality in mice due to congenital cardiovascular defects and *Rbpms* overexpression in smooth muscle cells (SMCs) in vivo can recapitulate differentiated alternative splicing status [47,48]. In the retina, RBPMS is identified as a specific neurochemical marker expressed in all RGCs throughout development and adulthood and is exclusively detected in RGCs [8,49,50,51]. The expression of RBPMS is also reliably used as a RGC-specific marker and persists in the remaining RGCs of RGC degeneration models in rats [52] and glaucoma and optic nerve crush injury models in mice [9,12,53,54,55,56]. Thus, *Rbpms* locus serves as an ideal candidate for driving RGC-specific gene expression in normal and disease retinas. In this study, we generated a tamoxifen-inducible *Rbpms*-CreER^T2^ mouse line by inserting a P2A-CreER^T2^ in-frame with the C-terminus of RBPMS. We showed that using self-cleaving P2A peptide from porcine teschovirus-1 fusion allowed the co-expression of RBPMS and CreER^T2^. Unlike the early postnatal lethality phenotype by P4 observed in *Rbpms*-null allele [47], the heterozygous and homozygous *Rbpms*-CreER^T2^ knock-in mice showed no overt defect and displayed normal retinal structure, visual function, and transcriptome. Importantly, the *Rbpms*-CreER^T2^ recombinase is sensitively inducible and exhibits a high recombination efficiency with the activation of the tdTomato reporter expression in nearly all adult RGCs, which makes this *Rbpms*-CreER^T2^ mouse line a valuable tool for studying gene function spatiotemporally in RGCs.

## 2. Materials and Methods

### 2.1. Mice and Tamoxifen Treatment

To generate the *Rbpms*-CreER^T2^ knock-in allele, we used the CRISPR/Cas9 genome editing method and single guide RNA (sgRNA), which targeted sequences 5′-AAGTCCCGGCAGTTCTGCTG-3′ (protospacer adjacent motif (PAM) site AGG), to insert a P2A-CreER^T2^ gene cassette as an in-frame fusion to the C-terminus of RBPMS. The sgRNA (synthesized by Synthego Corp., Redwood City, CA, USA), double-stranded P2A-CreER^T2^ repair template (synthesized by Azenta Life Sci., Burlington, MA, USA, Supplemental Figure S1A), and Cas9 protein (Alt-R^®^S. p. Cas9 Nuclease V3 from Integrated DNA Technologies, Inc., Coralville, IA, USA) were co-injected into C57BL/6J zygotes (Jackson Laboratory, Bar Harbor, ME, USA, Stock #009086). Founder mice were obtained and confirmed by long-range PCR genotyping and Sanger sequencing. The primer sets to verify the targeted allele were: 5′ external (Forward 5′-CGTCTTGGAGAAGCAACAGGGTCT-3′ and Reverse 5′-CCAGGTTCCTGATGTCCTGGCATCTGTC-3′, PCR amplicon of 742 bp), 3′ external (Forward 5′-GATCCTGGGCTGCCTGGTGCAAGCTGA-3′ and Reverse 5′-GCCTTCGGGTGTGACGTGTCCTGA-3′, PCR amplicon of 2072 bp), the CreER^T2^ internal (Forward 5′-GATCCTGGGCTGCCTGGTGCAAGCTGA-3′ and Reverse 5′-CCAGGTTCCTGATGTCCTGGCATCTGTC-3′, PCR amplicon of 335 bp), and *Rbpms* wild type (Forward 5′-CGTCTTGGAGAAGCAACAGGGTCT-3′ and Reverse 5′-GCCTTCGGGTGTGACGTGTCCTGA-3′, PCR amplicon of 347 bp). The correctly targeted founders were bred with C57BL/6J mice, and PCR genotyping and Sanger sequencing were again performed to confirm the germline transmission.

All animal experiments were performed in accordance with protocols approved by the Institutional Animal Care and Use Committee (IACUC, Protocol 2019–1012 Approved on 17 November 2022) at Augusta University following guidelines described in the US National Institutes of Health Guide for the Care and Use of Laboratory Animals. Mice were housed in a standard 12 h light/12 h dark cycle environment. Mice were treated with tamoxifen (75 mg/kg) by intraperitoneal (IP) injection at various developmental time points, and progesterone (50 mg/kg) was co-administered to prevent late-stage abortions in pregnant mice. Retinas were harvested 7 days after the final tamoxifen injection in adult mice and 2 days post-final tamoxifen injection at embryonic stages in pregnant mice or in P0 newborn pups. Embryonic day 0.5 was identified as the day when the vaginal plug was detected. The B6. Cg-Gt (ROSA) 26^Sortm14 (CAG-tdTomato) Hze/J^ Cre reporter mice [57] were purchased from the Jackson Laboratory (Strain #007914).

### 2.2. Histology and Immunohistochemistry

Mice were euthanized using carbon dioxide (CO_2_), followed by cervical dislocation. For hematoxylin and eosin (H&E) staining, whole eyeballs were embedded in the O.C.T compound (Tissue-Tek, Sakura Finetek, Torrance, CA, USA) directly. The frozen blocks were sectioned at a thickness of 7 um. Briefly, retinal frozen sections were immersed into hematoxylin for 5 min after fixation, followed by washing in ddH_2_O twice for 1 min each. Slides were then dipped in 0.5% Eosin for 45 s, then washed in ddH_2_O, dehydrated in graded ethanol, and vitrified by xylene. Slides were mounted with Permount^TM^ mounting medium (ThermoFisher Scientific Corp., Waltham, MA, USA, #SP15-100). Images for retinal structure were captured using a Nikon TE2000-U microscope with bright field and DIC (Differential Interference Contrast) optics.

Immunofluorescence staining was conducted as previously described [58]. Briefly, the eyeballs were dissected in cold-PBS as soon as possible; after the cornea and lens were removed, eyecups were fixed in 4% PFA for 30 min to 1 h. Eyecups were dehydrated within graded sucrose solutions until sunk in 30% sucrose totally and embedded in the OCT compound. Cryosections were cut at a thickness of 14 um. For immunostaining, retinal cryosections were washed 2 × 15 min in 0.1 M PBS (pH 7.2), followed by 2 × 15 min in 0.3% PBST (0.1 M PBS plus Triton X-100). The 10% normal horse serum in 0.3% PBST was applied to block non-specific binding sites for 1 h at room temperature. The primary antibodies were incubated overnight at 4 °C. After rinsing 4 × 10 min in 0.3% PBST, the corresponding secondary antibody was applied for 1.5 h at room temperature. For whole-mount retinas, whole eyeballs were extracted from the euthanized mice and fixed in 4% PFA overnight at 4 °C. On the following day, dissected retinas were washed and immunolabelled as above procedure after incubation in blocking buffer for about 2 h at room temperature, four incisions were made in retinas by fine scissor and flattened like a flower by cover slips. 4′,6-diamidino-2-phenylindole dihydrochloride (DAPI) (ThermoFisher Scientific #D1306) was used as a counterstain to label nuclei before mounting slides. The primary antibodies used were guinea pig anti-RBPMS (1:500, Phospho-Solutions LLC, Aurora, CO, USA, #1832), rabbit anti-RFP (1:1000, Rockland Immunochemicals, Inc., Pottstown, PA, USA, #600-401-379), rat anti-RFP (1:1000, ChromoTek, ProteinTech Group, Rosemont, IL, USA, #5F8), rabbit anti-CRE (1:1000, Cell Signaling Technology, Danvers, MA, USA, #15036), mouse anti-POU4F1 (1:500, Santa Cruz Biotechnology (SCBT), Inc., Dallas, TX, USA #SC-8429), goat anti-POU4F2 (1:200, SCBT, #SC-6026), and rabbit anti-CDKN1B/p27Kip1 (1:200, Abcam PLC, Cambridge, UK, #ab32034). Corresponding secondary antibodies were applied at a dilution of 1:1000 were: Alexa-488-conjugated donkey anti-guinea pig (Jackson Immuno Research Labs, West Grove, PA, USA, #NC0194368), Alexa-568-conjugated donkey anti-rabbit (ThermoFisher Scientific #A-10042), Alexa-568-conjugated goat anti-rat (ThermoFisher Scientific #A-11077), Alexa-647-conjugated donkey anti-mouse (ThermoFisher Scientific #A-32787), Alexa-647-conjugated donkey anti-goat (ThermoFisher Scientific #A-32849).

### 2.3. Optomotor Response

Visual acuity and contrast sensitivity were evaluated using the OptoMotry device from Cerebral Mechanics (Medicine Hat, Alberta, AB, Canada). Briefly, the individual mouse was placed on the central platform inside the chamber, and computer monitors surrounding the mouse projected a virtual stimulus in the form of a sine wave. Animals tracked the grating with head and neck movements in the direction of grid rotation clockwise or counterclockwise. The system measured visual function in each eye separately because motion in the temporal to the nasal direction of either eye elicited a tracking response. A camera above monitored the behavior of the mouse, allowing the rater to detect the response in corporal and give a score of yes or no. To examine visual acuity, spatial frequency thresholds (cycles per degree) were measured by systematically increasing the spatial frequency of the grating (decreasing the bar width) at full contrast until mice could no longer track. The rotation speed was fixed at 12 degrees per second, and the data were managed and generated by the software. Data are presented as responses from both eyes.

### 2.4. Fluorescein Angiography

Fluorescein Angiography (FA) was used to analyze the retinal vasculature. Mice were anesthetized by IP injection with Ketamine/Xylazine cocktail (Ketamine 85 mg/cc, Xylazine 10 mg/cc) at a dosage of 1 uL/g body weight. The pupils were dilated with 1% tropicamide (Akorn, Lake Forest, IL, USA) and the corneas were moisturized with GenTeal Systane lubricant eye drops (Alcon, Ft. Worth, TX, USA). Fluorescein injection solution (Apollo Ophthalmic, 10%, Newport Beach, CA, USA) was administered by IP injection, and retinas were imaged with the Micron IV system (Phoenix Laboratories, Bend, OR, USA) 3–5 min later.

### 2.5. Electroretinography

Full-field electroretinography (ffERG) and pattern electroretinography (PERG) were used to evaluate the overall retinal circuits and targeted RGC function, respectively. The procedures were blindly conducted as previously described [59]. Briefly, mice were dark-adapted overnight (>12 h) and anesthetized by IP injection with Ketamine/Xylazine cocktail as described above. The pupils were dilated using 0.5% tropicamide and 2.5% phenylephrine, and 0.3% hypromellose (GenTeal Systane lubricant eye drops, Alcon, Ft. Worth, TX, USA) was applied to the ERG probes as well as the cornea, supplying the moisture of the cornea and a lubricating cushion between the cornea and testing probe. The procedure was conducted with the Celeris Ophthalmic Electrophysiology System (Diagnosys LLC, Lowell, MA, USA). Experiments consisted of a series of scotopic tests with 5 ms flashes of increasing luminance, followed by photopic testing with 5 and 500 ms flashes above a pedestal, and the photopic stimuli included a “natural” noise stimulus. For PERG test, the dark-adapted mice were anesthetized as described for ffERG prior to the examination. The pattern stimulator was placed in contact with one eye while the flash stimulator in contact with the contralateral eye, acting as the reference electrode. The parameters for PERG amplitudes were set under the spatial frequency of 0.125 cycles/degree, a luminance of 50 cd.s/m^2^, contrast of 100%, and substantial averaging (600 sweeps). The transient PERG responses were recorded using the Celeris system following the manufacturer’s guidelines. All ERG data were analyzed by the software Espion V6 (Diagnosys LLC). The P1-N2 amplitudes, measured from the P1 peak to the nadir of N2, were additionally used to analyze the RGC function.

### 2.6. Optical Coherence Tomography 

Spectral domain-optical coherence tomography (SD-OCT) was performed to assess the retinal structure. Mice were anesthetized as described above. Anesthetized mice were wrapped in soft tissue paper and placed within the mouse-holding cassette. To maintain the moisture and clarity of the cornea, 0.3% hypromellose eye gel and 0.4% polyethylene glycol 400 (Systane Ultra lubricating eye drops, Alcon, Ft. Worth, TX, USA) were applied during the entire procedure. The SD-OCT images were obtained with the Bioptigen Spectral Domain Ophthalmic Imaging System (SDOIS, Bioptigen, Morrisvillle, NC, USA, # Envisu R2200). Imaging included averaged single B scan and volume intensity scans with images centered on the optic nerve head. InVivoVueTM Diver 2.4 software (Bioptigen) was used for image analysis and auto-segmentation report analysis. As the auto-segmentation reflects average retinal layer thickness, the manual segmentation analysis was also conducted to precisely reveal the thickness of the retinal nerve fiber layer (RNFL) and inner plexiform layer (IPL). Data for a given retinal layer in each group were averaged.

### 2.7. RNA-Sequencing (RNA-Seq)

Three repeats were performed for the strand-specific RNA-Seq experiments with retinas from mice at P42, and a pair of retinas were collected as one sequencing sample. The flash frozen retinas in Trizol were shipped to Azenta Life Science (South Plainfield, NJ, USA) to perform RNA extraction and library preparation. Briefly, total RNA extraction was carried out using Trizol following manufacturer’s instructions (ThermoFisher Scientific). The strand-specific RNA sequencing library was prepared using NEBNext Ultra II Directional RNA Library Prep Kit for Illumina following manufacturer’s instructions (New England Biolabs, Inc., Ipswich, MA, USA), and PolyA selection for mRNA was used to remove rRNA. Sequencing was performed on the Illumina HiSeq 4000, and the depth was 20–30 million reads per sample. The Trimmomatic software (github.com/*usadellab*/Trimmomatic) was used to filter and trim minority low quality sequencing reads from the data set. The quality of sequence reads in FASTQ files was evaluated using the FastQC analysis. High quality sequence reads were aligned to the mouse mm10 reference transcriptome using the HISAT2. The HISAT2 outputs were transformed with SAMtools and then fed into StringTie for transcripts quantification. Subsequently, the count tables or matrices were input into the DESeq2 statistical package for determination of differential transcript expression between samples (cutoff fold change ≥ 2 and adjusted *p* value ≤ 0.05).

### 2.8. Quantification and Statistics Analysis

Cell counts were performed manually with ImageJ. Cell counts were blindly conducted on H&E-stained sections of an area of 250 μm × 250 μm, or 290 μm × 290 μm area of immuno-stained sections in central retinas. Nasal and temporal retinal areas beginning 200 μm from the optic nerve were analyzed. For each group, at least three animals were tested without preferentially considering subject sex. Within GraphPad Prism 9.0 (GraphPad, La Jolla, CA, USA), data were analyzed by one-way ANOVA or two-way ANOVA for comparing single parameters or two and more factors respectively. All values reported in this study present the mean ± SEM. The significance was identified as *p* < 0.05.

## 3. Results

### 3.1. Generation and Identification of Rbpms-CreER^T2^ Mice

**Figure 1:**
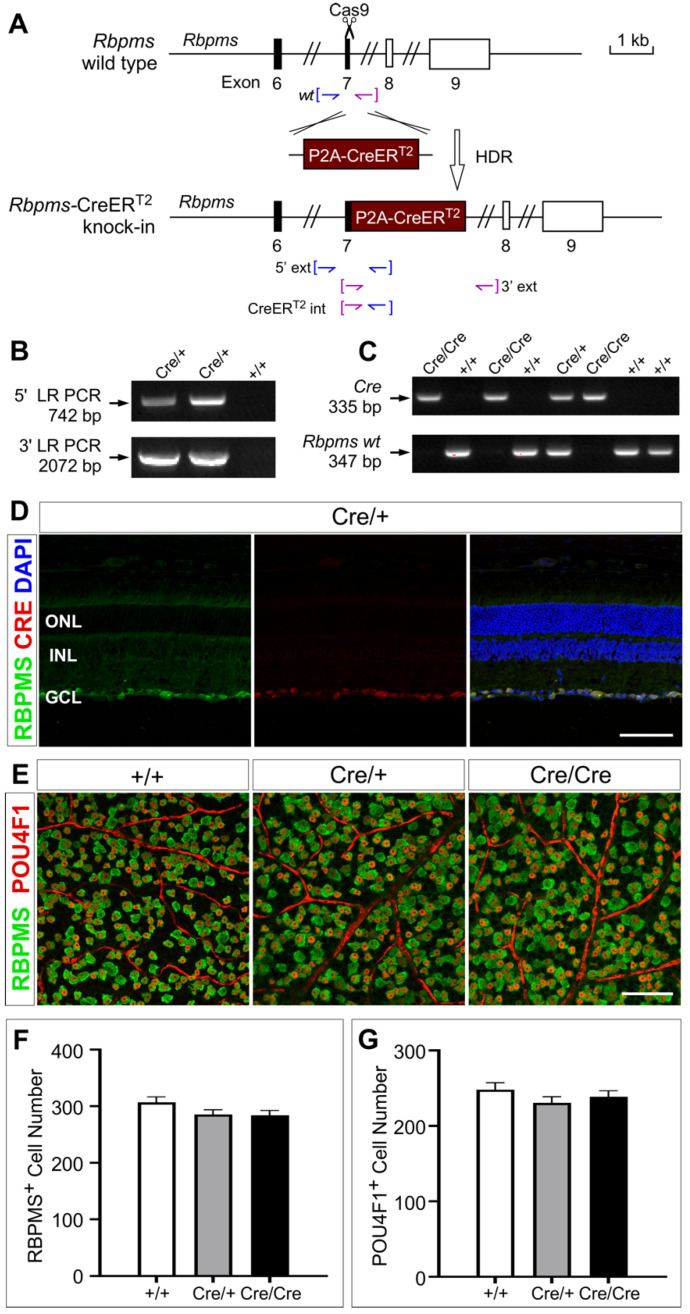
Generation and confirmation of *Rbpm*s-CreER^T2^ mice. (**A**) *Rbpms*-CreER^T2^ targeting strategy by fusing P2A-CreER^T2^ sequence in-frame to the C-terminus of *Rbpms*. (**B**) Representative long-range PCR amplification results show the 742 bp external fragment at 5′ and the 2072 bp amplicon at 3′ of knock-in sequence, confirming the correct allele using 5′ and 3′ external primer sets, respectively. (**C**) Representative PCR genotyping of a litter from crossing of *Rbpms^CreERT2/+^* mice identify a 335 bp Cre fragment and a 347 bp wild type fragment, confirming the correct targets using *Cre* and *Rbpms wt* primers, respectively. (**D**) Co-labeling with anti-RBPMS and anti-CRE antibodies in 6 weeks retinas show the overlapping expression of CreER^T2^ and endogenous RBPMS. (**E**) Immunostaining with antibodies against RBPMS and POU4F1 of wild type, *Rbpms^CreERT2/+^* and *Rbpms^CreERT2/CreERT2^* retinal whole-mounts show the similar expression of RBPMS and POU4F1 in the ganglion cell layer (GCL). (**F**,**G**) Quantification of the RBPMS^+^ (**F**) and POU4F1^+^ (**G**) cells reveals no significantly difference among wild type, *Rbpms^CreERT2/+^* and *Rbpms^CreERT2/CreERT2^* retinas. *n* = 3. Statistical significance was assessed by one-way ANOVA followed by Tukey’s multiple comparisons test. ONL and INL denote outer nuclear layer and inner nuclear layer, respectively. Scale bars equal 50 μm.

To develop an RGC-specific inducible Cre mouse line, we chose to insert a P2A-CreER^T2^ gene cassette in in-frame fusion to the C-terminus of RBPMS, an RNA splicing protein known to be expressed in all RGCs (Figure 1A). The addition of the highly efficient P2A self-cleaving peptide allows the co-expression of RBPMS and CreER^T2^ from a single transcript due to ribosomal skipping during protein translation [60] without disrupting the function of RBPMS. CreER^T2^, a Cre recombinase fused to a mutant human estrogen ligand-binding domain, permits the nuclear translation and activation of Cre recombinase activity upon binding of synthetic ligands, such as 4-hydroxytamoxifen and tamoxifen [61].

We used genome editing with CRISPR/Cas9 approach and performed cytoplasmic microinjection of C57BL/6J zygotes with a mixture of sgRNA, repair template, and Cas9 protein. The correctly targeted *Rbpms*-CreER^T2^ founder mice were confirmed by PCR genotyping using external primers and Sanger sequencing (Figure 1B, Supplemental Figure S1B). The offspring generated from the correct founder crossed with C57BL/6J were further verified by PCR genotyping and Sanger sequencing (Figure 1C, Supplemental Figure S1B).

To test whether the expression of knock-in CreER^T2^ recapitulated the RGC-specific expression pattern of the endogenous RBPMS, co-immunolabeling with anti-RBPMS and anti-CRE antibodies was performed and detected the expression of both RBPMS and CreER^T2^ specifically in the RGCs of adult heterozygous *Rbpms^CreERT2/+^* (also labeled as Cre/+) retinas (Figure 1D). RBPMS expression overlapped that of CreER^T2^, revealed by anti-CRE, indicating that the endogenous *Rbpms* locus faithfully directs the expression of CreER^T2^ in all RBPMS-expressing RGCs in adult retinas.

A recent study shows that inactivation of *Rbpms* results in perinatal lethality in mice due to congenital cardiovascular defects [47]. Hence, we tested whether, in *Rbpms*-CreER^T2^ mice, the insertion of a P2A-CreER^T2^ in-frame with the C-terminus of RBPMS led to the co-expression of RBPMS and CreER^T2^, thus avoiding the disruption of the endogenous *Rbpms*. We intercrossed heterozygous *Rbpms^CreERT2/+^* mice to generate homozygous *Rbpms^CreERT2/CreERT2^* (also labeled as Cre/Cre) mice and evaluated the basic physiological characteristics in the knock-in alleles. We observed that both heterozygous *Rbpms^CreERT2/+^* mice and homozygous *Rbpms^CreERT2/CreERT2^* mice were viable and fertile, exhibiting no overt abnormality in body weight and eye size (Supplemental Figure S1C–E). To investigate whether the *Rbpms*-CreER^T2^ knock-in allele had a detrimental effect on RGCs, we further performed co-immunostaining of whole-mount retinas with anti-RBPMS and anti-POU4F1 antibodies, which mark all and about 80% RGCs, respectively [49,50,51,62]. We counted about 40 independent images for each group of the wild type control (+/+), *Rbpms^CreERT2/+^* and *Rbpms^CreERT2/CreERT2^* retinas. The results showed that there was no significant difference in the number of RBPMS^+^ and POU4F1^+^ RGCs in the GCL among these mice (Figure 1F,G, Supplemental Table S1). Moreover, the expression of RBPMS in RGCs was similar in number of RGCs as well as signal intensity in wild type, *Rbpms^CreERT2/+^* and *Rbpms^CreERT2/CreERT2^* retinas (Figure 1E), suggesting that the fusion of P2A-CreER^T2^ to the C-terminus of RBPMS should have no impact on the expression of RBPMS. Overall, our data demonstrated that this novel *Rbpms*-CreER^T2^ knock-in mouse line allows the co-expression of RBPMS and CreER^T2^ specifically in all RGCs without overt phenotypes.

### 3.2. Assessment of Retinal Feature and Transcriptome in Rbpms-CreER^T2^ Knock-in Mice 

**Figure 2:**
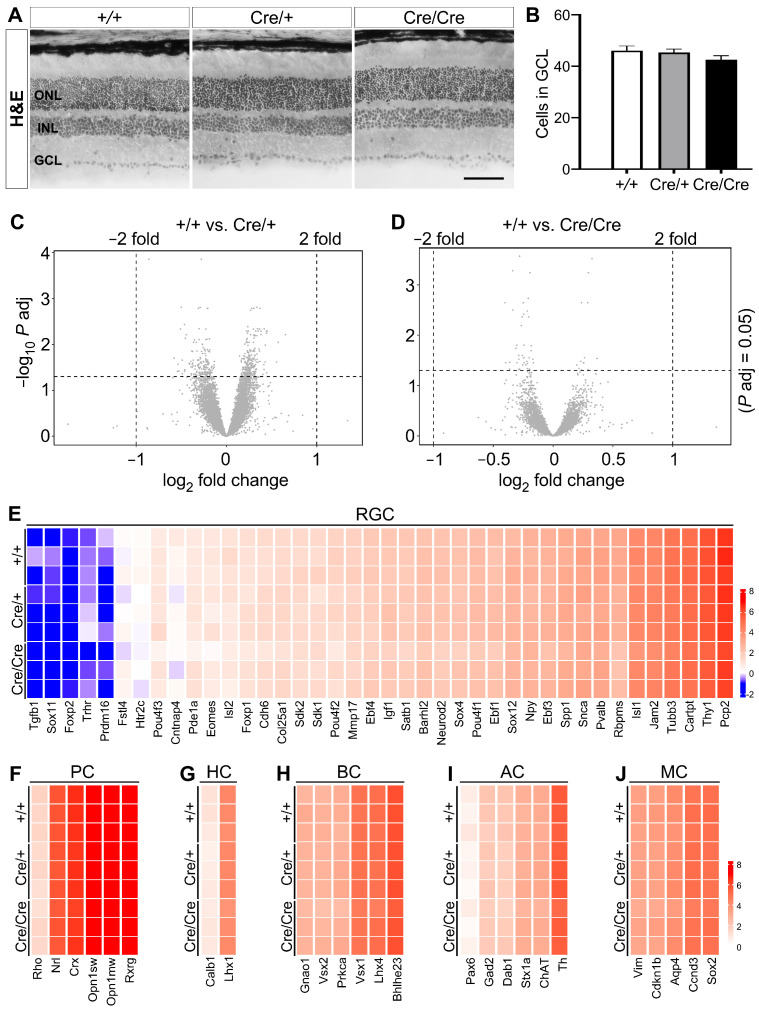
*Rbpms*-CreER^T2^ knock-in does not alter retinal structure and transcriptome. (**A**) Representative images of H&E stained retinal cryosections reveal comparable retinal cellular structure among the wild type, *Rbpms^CreERT2/+^* and *Rbpms^CreERT2/CreERT2^* mice. (**B**) Quantification of cell number in the ganglion cell layer (GCL) within 250 µm × 250 µm region shows no significant changes among the wild type, *Rbpms^CreERT2/+^* and *Rbpms^CreERT2/CreERT2^* mice. (**C**,**D**) Volcano plots display the RNA-Seq results. Note that using the criteria of adjusted *p* < 0.05 and fold change > 2, no differentially expressed gene (DEG) with significant change is identified (**C**) between wild type and *Rbpms^CreERT2/+^* retinas and (**D**) between wild type and *Rbpms^CreERT2/CreERT2^* retinas. (**E**–**J**) Heatmaps show the expression level of selected genes expressed in individual retinal cell types. Note that there is no significant difference among the control, *Rbpms^CreERT2/+^* and *Rbpms^CreERT2/CreERT2^* retinas of in the expression of marker genes specific for (**E**) retinal ganglion cell (RGC), (**F**) photoreceptor cell (PC), (**G**) horizontal cell (HC), (**H**) bipolar cell (BC), (**I**) amacrine cell (AC), and (**J**) Müller glial cell (MC). The cutoff criteria are set as adjusted *p* < 0.05 and fold change > 2. *n* = 3. Statistical significance was assessed by one-way ANOVA followed by Tukey’s multiple comparisons test. ONL indicates outer nuclear layer, INL and GCL denote inner nuclear layer and ganglion cell layer, respectively. Scale bar equals 50 μm.

To evaluate whether the retinal structure and transcriptome was influenced in *Rbpms*-CreER^T2^ knock-in mice, we first carried out hematoxylin and eosin (H&E) staining of retinal sections of wild type, *Rbpms^CreERT2/+^* and *Rbpms^CreERT2/CreERT2^* mice at P42 and detected no change in the overall retinal structure or in each of the three cellular layers (Figure 2A). The GCL contains not only RGCs, but also displaced amacrine cells and astrocytes [63,64,65,66]. Quantification of the number of cells in the GCL revealed no significant difference among the three groups nor any significant change in the number of cells in the INL and ONL (Figure 2B, Supplemental Table S1).

Additionally, we performed the bulk RNA-Seq of whole retinas at P42 to assess any possible changes in transcriptome caused by P2A-CreER^T2^ knock-in alleles. The volcano maps generated by the entire DEG lists displayed no significant change in the expression of genes meeting the standard criteria (fold change > 2, adjusted *p* < 0.05) in both *Rbpms^CreERT2/+^* and *Rbpms^CreERT2/CreERT2^* retinas (Figure 2C,D). A comparison of the expression levels determined by RNA-Seq revealed no significant difference among control, *Rbpms^CreERT2/+^,* and *Rbpms^CreERT2/CreERT2^* retinas of selective marker genes in RGCs (Figure 2E) as well as in other retinal cell types such as photoreceptor cells, horizontal cells, bipolar cells, amacrine cells, and Müller glial cells (Figure 2F–J). Among these are genes known to be specific to RGCs, such as *Pou4f1*, *Thy1*, *Snca*, and *Rbpms*. Together, these results suggest that *Rbpms*-CreER^T2^ knock-in might have no significant impact on the transcriptome of RGCs as well as of other retinal cell types.

### 3.3. Assessment of Retinal Function in Rbpms-CreER^T2^ Knock-in Mice

**Figure 3:**
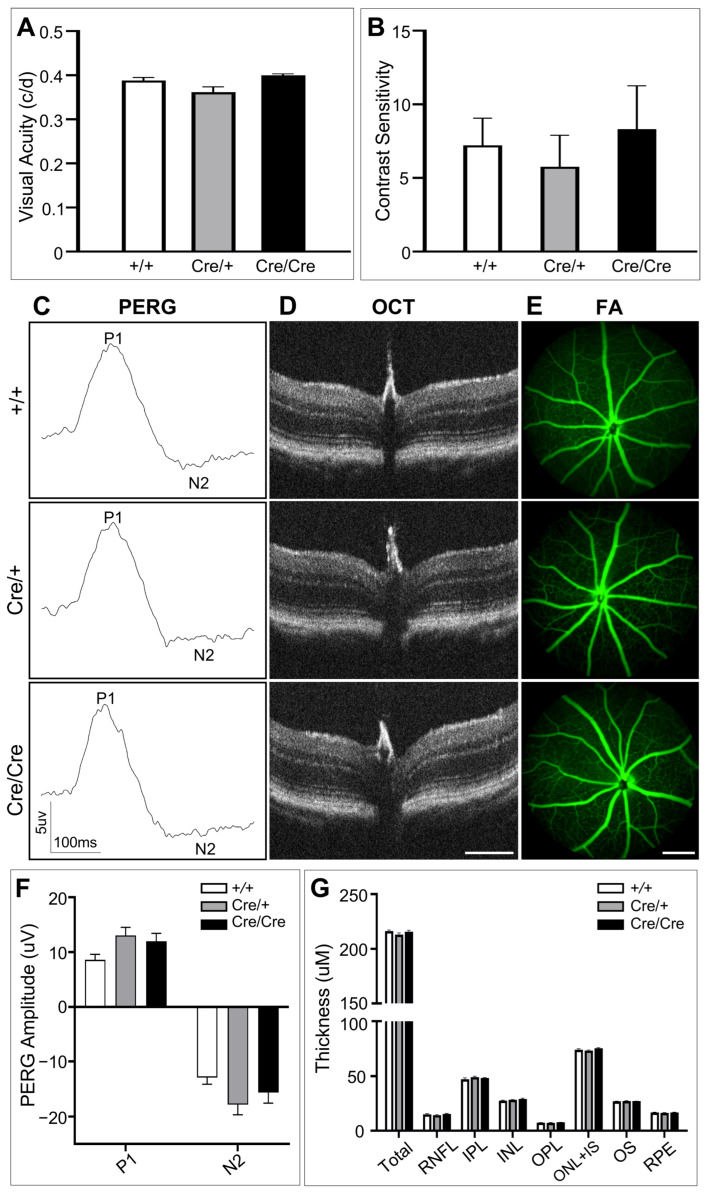
Analysis of *Rbpms*-CreER^T2^ knock-in effects on visual function and retinal fundus features. (**A**,**B**) Optomotor response measurements show no significant changes in (**A**) visual acuity and (**B**) contract sensitivity among wild type, *Rbpms^CreERT2/+^* and *Rbpms^CreERT2/CreERT2^* mice. (**C**) Pattern electroretinography (PERG) was performed on the same mice with the 50 cd.s/m^2^ “natural” stimulus, averaged amplitude of P1 and N2 components from representative wild type, *Rbpms^CreERT2/+^* and *Rbpms^CreERT2/CreERT2^* mice does not exhibit overt differences between each of the two groups. (**D**) Quantification of amplitudes of P1 and N2 in PERG. (**E**,**F**) Representative optical coherence tomography (OCT) images (**E**) and measurement of each layer thickness evaluated by OCT (**F**) show no significant difference among wild type, *Rbpms^CreERT2/+^* and *Rbpms^CreERT2/CreERT2^* mice. (**G**) Fluorescein angiography (FA) examination reveals comparable retinal vessel features among wild type, *Rbpms^CreERT2/+^* and *Rbpms^CreERT2/CreERT2^* mice. *n* = 4. Each bar represents the mean ± SEM. Statistical significance was assessed by one-way ANOVA followed by Tukey’s multiple comparisons test (**D**) or two-way ANOVA (**F**). Scale bars equal 300 μm.

Visual acuity (VA) is frequently used to test the ability to resolve fine details while the ability to distinguish objects against the background is assessed by contrast sensitivity (CS) [67]. We first measured the binocular VA and CS of mice at 6–8 weeks of age by optomotor reflex (OMR). Compared to the wild type mice, no significant change was detected by VA tests of *Rbpms^CreERT2/+^* and *Rbpms^CreERT2/CreERT2^* mice (mean ± SEM: Cre/+ vs. *+/+* = 0.372 ± 0.02 vs. 0.389 ± 0.017, *p* = 0.18; Cre/Cre vs. *+/+* = 0.395 ± 0.007 vs. 0.389 ± 0.017, *p* = 0.76; *n* = 4) (Figure 3A). Similarly, CS measurement did not detect any significant change in heterozygous and homozygous *Rbpms*-CreER^T2^ knock-in mice compared to the wild type controls (mean ± SEM: Cre/+ vs. +/+ = 5.77 ± 5.65 vs. 7.217 ± 4.89, *p* = 0.9; Cre/Cre vs. +/+ = 8.324 ± 7.74 vs. 7.217 ± 4.89, *p* = 0.94; *n* = 4) (Figure 3B).

Moreover, to test whether RGC-specific visual function was disrupted in *Rbpms*-CreER^T2^ knock-in mice, we employed pattern electroretinography (PERG) analysis that is a non-invasive, direct, and objective method mostly assessing RGCs function. The results showed that two main components in PERG, i.e., amplitudes of positive wave P1 and negative wave N2 at 50 cd.s/m^2^, were not notably different between wild type and *Rbpms^CreERT2/+^* mice or between wild type and *Rbpms^CreERT2/CreERT2^* mice (P1 amplitudes mean ± SEM: Cre/+ vs. +/+ = 13.04 ± 3.83 vs. 8.58 ± 2.68, *p* = 0.07; Cre/Cre vs. +/+ = 11.95 ± 3.89 vs. 8.58 ± 2.68, *p* = 0.19; *n* = 4) (N2 amplitudes mean ± SEM: Cre/+ vs. +/+ = −17.82 ± 4.86 vs. −12.87 ± 3.27, *p* = 0.13; Cre/Cre vs. +/+ = −15.59 ± 5.24 vs. −12.87 ± 3.27, *p* = 0.51; *n* = 4) (Figure 3C,D), demonstrating that P2A-CreER^T2^ knock-in does not impact the function of RGCs in both heterozygous and homozygous *Rbpms*-CreER^T2^ knock-in mice.

We further used spectral domain optical coherence tomography (SD-OCT) and fluorescein angiography (FA) to investigate the retinal architecture and vessels, respectively. Compared with the wild type mice, the retinal structure of *Rbpms^CreERT2/+^* and *Rbpms^CreERT2/CreERT2^* mice remained unchanged with comparable thickness across all layers (Figure 3E,F). FA also showed similar vascular distribution and minimal vessel tortuosity among wild type, heterozygous, and homozygous *Rbpms-*CreER^T2^ knock-in retinas (Figure 3G), confirming that the fundus feature and retinal laminar structure were unaffected in the knock-in mice.

**Figure 4:**
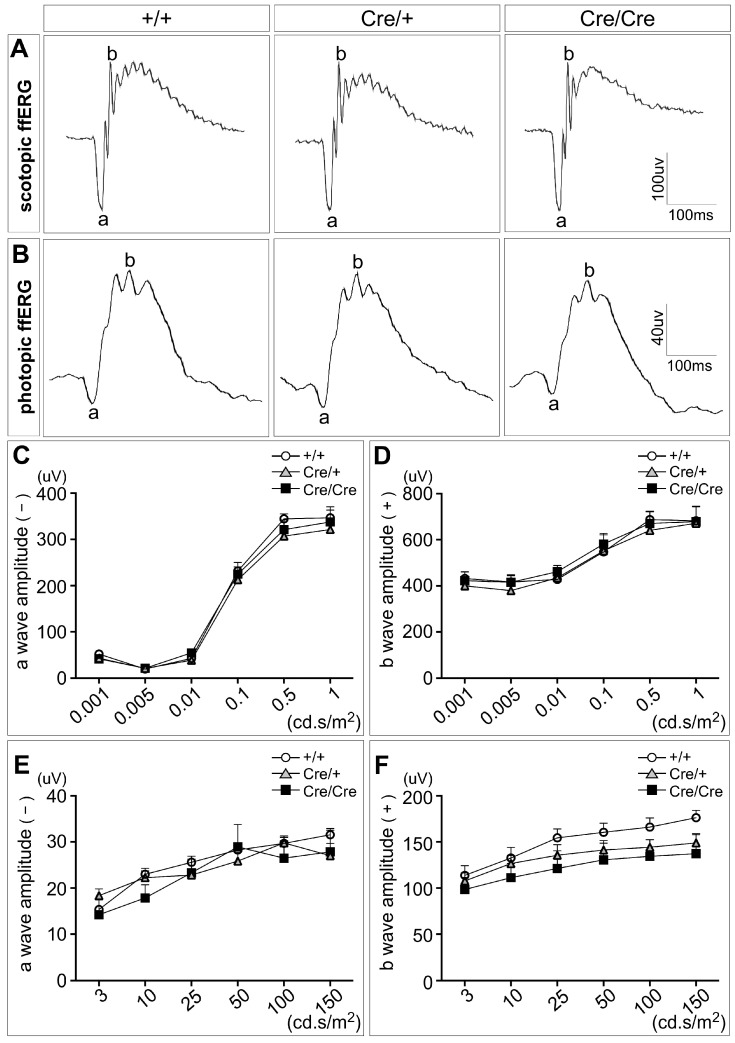
Assessment of *Rbpms*-CreER^T2^ knock-in effects on retinal function. (**A**) Representative scotopic ERG waveforms from the wild type, *Rbpms^CreERT2/+^,* and *Rbpms^CreERT2/CreERT2^* mice at P42. (**B**) Representative photopic ERG waveforms from the same mice tested after scotopic ERG. (**C**,**D**) Summary of luminance-response functions for the dark-adapted ERG (**C**) a-wave and (**D**) b-wave. Two-way ANOVA test revealed that heterozygous and homozygous *Rbpms*-CreER^T2^ knock-in has no significant effect on either a-wave (*F*_10, 54_ = 0.36, *p* = 0.96) or b-wave (*F*_10, 54_ = 0.11, *p* = 1.0). (**E**,**F**) Summary of luminance-response functions for the light-adapted ERG (**E**) a-wave and (**F**) b-wave. No significant difference is seen among the wild type, *Rbpms^CreERT2/+^,* and *Rbpms^CreERT2/CreERT2^* mice based on two-way ANOVA analysis of a-wave (*F*_10, 54_ = 0.53, *p* = 0.86) and b-wave (*F*_10, 54_ = 0.15, *p* = 1.0). *n* = 4. Data points represent the mean ± SEM.

Additionally, to further assess the effect of *Rbpms*-CreER^T2^ knock-in on retinal function, we performed full-field electroretinography (ffERG) experiments in wild type, *Rbpms^CreERT2/+^,* and *Rbpms^CreERT2/CreERT2^* mice at P42. We first compared the scotopic a-waves, which mostly reflect the response from photoreceptors, and the scotopic b-waves, which primarily detect a rod response from bipolar cells, and found that neither amplitudes of the negative a-waves provoked by photoreceptors nor the positive b-waves driven by bipolar cells exhibited any distinguishable alternations in *Rbpms^CreERT2/+^* and *Rbpms^CreERT2/CreERT2^* mice under dark-adapted conditions (Figure 4A,C,D, Supplemental Table S2). Under light-adapted conditions, the photopic a-waves were comparable among wild type, *Rbpms^CreERT2/+^,* and *Rbpms^CreERT2/CreERT2^* mice while there was a small but insignificant decrease in b-waves of *Rbpms^CreERT2/+^* and *Rbpms^CreERT2/CreERT2^* mice (Figure 4B,E,F, Supplemental Table S3). Taken together, our data demonstrate that the retinal structure and function are unaffected in both heterozygous and homozygous *Rbpms*-CreER^T2^ mice.

### 3.4. Assessment of the Cre Recombinase Activity and Efficiency in Activating Reporter Gene Expression

**Figure 5:**
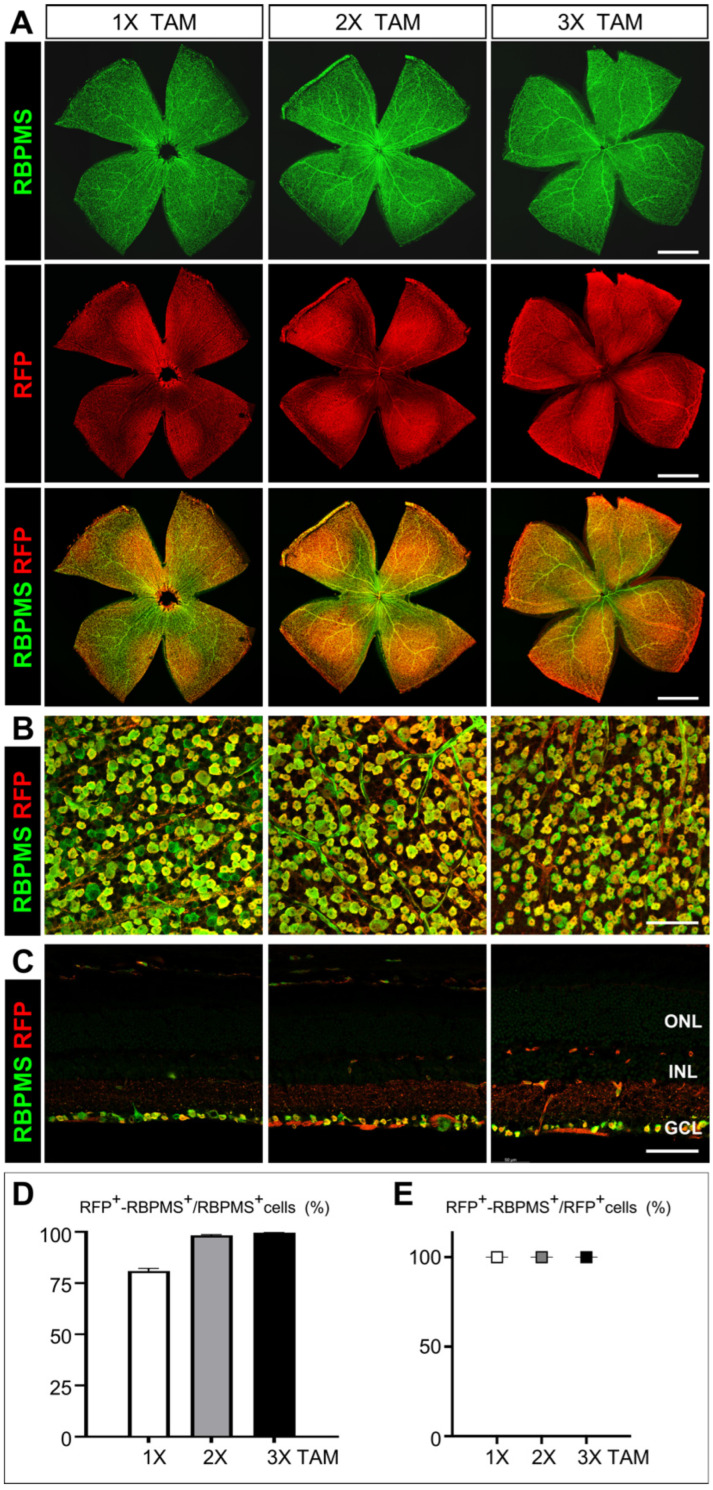
Assessment of Cre recombinase activity in RGCs of *Rbpms*-CreER^T2^ knock-in mice. (**A**) Representative images of flat-mount retinas of 8-week-old *Rbpms^CreERT2/+^*; *Rosa26^td^*^T/+^ mice. One to three dosages of tamoxifen were administered starting at P50 and retinas were harvested 7 days later for immunolabeling with antibodies against RBPMS (green) and RFP (red) to detect tdTomato. (**B**) Representative enlarged views of flat-mount retinas in (**A**). (**C**) Representative images of immunolabeled retinal cryosections of 8-week-old *Rbpms^CreERT2/+^*; *Rosa26^td^*^T/+^ mice with one to three dosages of tamoxifen. (**D**) Quantification of *Rbpms*-CreER^T2^ recombinase efficiency and (**E**) Quantification of *Rbpms*-CreER^T2^ recombinase specificity. The CreER^T2^-mediated recombination efficiency was calculated as the percentage of tdTomato^+^-RBPMS^+^/total RGCs positive for RBPMS, the CreER^T2^-mediated recombination specificity was calculated as the percentage of tdTomato^+^-RBPMS^+^/total tdTomato positive RGCs driven by RBPMS. *n* = 3. ONL indicates outer nuclear layer; INL and GCL denote inner nuclear layer and ganglion cell layer, respectively. The scale bars in (**A**) equal 1 mm while those in (**B**,**C**) equal 50 μm.

To determine the RGC-specificity and efficiency of Cre recombinase-mediated recombination in *Rbpms*-CreER^T2^ mice, we crossed *Rbpms*-CreER^T2^ mice with B6. Cg-*Gt* (*ROSA*)*26Sor^tm14(CAG-tdTomato)Hze^*/J tdTomato reporter (*Rosa26^tdT^*) strain to generate double heterozygous *Rbpms^CreERT2/+^*; *Rosa26^tdT/+^* mice. To activate CreER^T2^ recombinase and induce the expression of tdTomato, one to three consecutive daily dosages of tamoxifen at 75 mg/kg bodyweight were IP administered into *Rbpms^CreERT2/+^*; *Rosa26^tdT/+^* mice starting at P50, and the retinas were collected for immunolabeling 7 days after the final tamoxifen injection. Both retinal flat whole-mounts and cryosections were immunolabeled with anti-RBPMS, and anti-RFP antibodies and the ratios of tdTomato-RBPMS co-expressing RGCs (tdT-RBPMS double positive) to total RGCs (RBPMS-positive) were calculated to assess the efficiency of Cre recombination. The ratios of tdTomato-RBPMS co-expressing RGCs (tdT-RBPMS double positive) to all RBPMS-driven recombination RGCs (tdT-positive) were calculated to assess the specificity. Compared to the absence of tdTomato expression in uninduced mice (Supplemental Figure S2), a single dosage of tamoxifen resulted in about 80.98% RBPMS^+^ RGCs expressing tdTomato in whole mount retinas and 80.13% in retinal cryosections, demonstrating a high efficiency of Cre-loxP recombination driven by *Rbpms*-CreER^T2^ (Figure 5A–C left panels and D). With two consecutive tamoxifen dosages, the immunolabeling results showed that *Rbpms*-CreER^T2^ could activate the expression of tdTomato in most RGCs (98.28% in whole-mounts and 95.9% in cryosections) (Figure 5A–C middle panels and D). Three consecutive daily tamoxifen dosages further increased *Rbpms*-CreER^T2^ recombinase activity and introduced the activation of tdTomato in nearly all RGCs (99.61% in whole-mounts and 97.93% in cryosections) (Figure 5A–C right panels and D). In addition, all tdT-positive cells were found strictly co-expressing RBPMS with different dosages of tamoxifen, from one to three consecutive injections (Figure 5E). Taken together, the Cre recombinase activities of *Rbpms*-CreER^T2^ mice are highly and sensitively inducible in the adult retina, and the recombination is specifically restricted to the RGCs.

**Figure 6:**
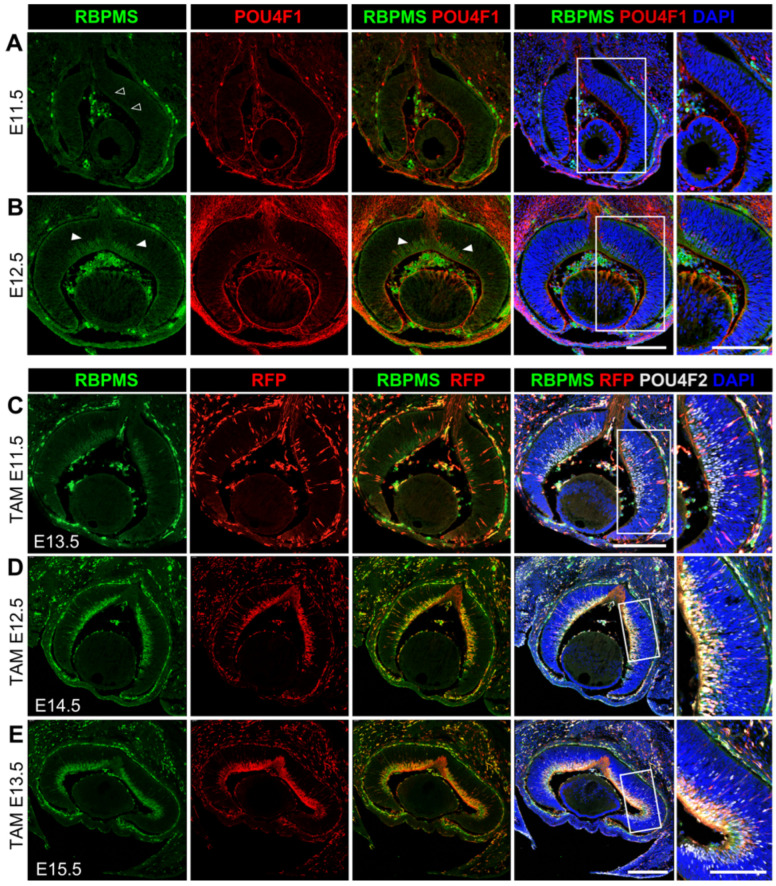
Characterization of *Rbpms*-CreER^T2^ recombinase activity during early retinal development. (**A**,**B**) Determination of the onset of RBPMS expression in RGCs. Cryosections of wild type developing retinas at (**A**) E11.5 and (**B**) E12.5 were immunolabeled with anti-RBPMS (green) and anti-POU4F1 (red) and nuclear counterstained with DAPI (blue). Arrowheads indicate RBPMS expression and RBPMS-POU4F1 co-expression in the central retina ((**B**), left and central panels, respectively). Far right panels show the enlarged views of the boxed regions. (**C**–**E**) Representative images of retinal cryosections immunolabeled for RBPMS, tdTomato (RFP) and POU4F2 after induction by a single dosage of tamoxifen. IP injection of tamoxifen was administered at (**C**) E11.5, (**D**) E12.5, and (**E**) E13.5, and retinas were collected for immunolabeling 2 days after tamoxifen induction at E13.5, E14.5, and E15.5, respectively. DAPI (blue) was used to stain the nuclei. Far right panels show the enlarged views of the boxed regions. Scale bars equal 250 μm.

Published studies have shown that during retinogenesis, the expression of *Rbpms* is detected in developing RGCs at E14.5 [68], we thus assessed the expression of *Rbpms* in more details and evaluated *Rbpms*-CreER^T2^ recombinase activities during RGC development and compared them with the spatiotemporal expression pattern of RBPMS in wild type retinas by immunostaining. While there was no detectable RBPMS expression in the developing retinas at E11.5 (Figure 6A), a few RBPMS-positive cells appeared in the central retina at E12.5 and also expressed POU4F1 (Figure 6B), suggesting that the onset of RBPMS expression should start shortly after the RGC differentiation and were restricted to RGCs in the developing retina.

We then determined the onset of *Rbpms*-CreER^T2^ recombinase activity and evaluated its cell type specificity and recombination efficiency during early retinal development. We delivered a single dosage of tamoxifen by IP injection into pregnant *Rbpms^CreERT2/+^*; *Rosa26^tdT/+^* mice at E11.5, E12.5, and E13.5, respectively, and harvested the eyes two days after IP injection for immunolabeling analysis with antibodies against RBPMS, RFP, and POU4F2. POU4F2 is one of the key transcription factors controlling RGCs development, and the onset of POU4F2 expression in retinas is one day earlier than POU4F1 in all RGCs, including nascent RGCs [28,69]. As seen in the immunostaining results, though RBPMS expression was not detectable at E11.5, administering tamoxifen at E11.5 was able to activate tdTomato expression in POU4F2-positive RGCs (Figure 6C). Administering tamoxifen at E12.5 and E13.5 elicited strong tdTomato expression in significantly more RGCs marked by POU4F2 and RBPMS (Figure 6D,E). These observations indicate that *Rbpms*-CreER^T2^ recombinase activity is present in the nascent RGCs to drive a high efficiency of Cre recombination.

**Figure 7:**
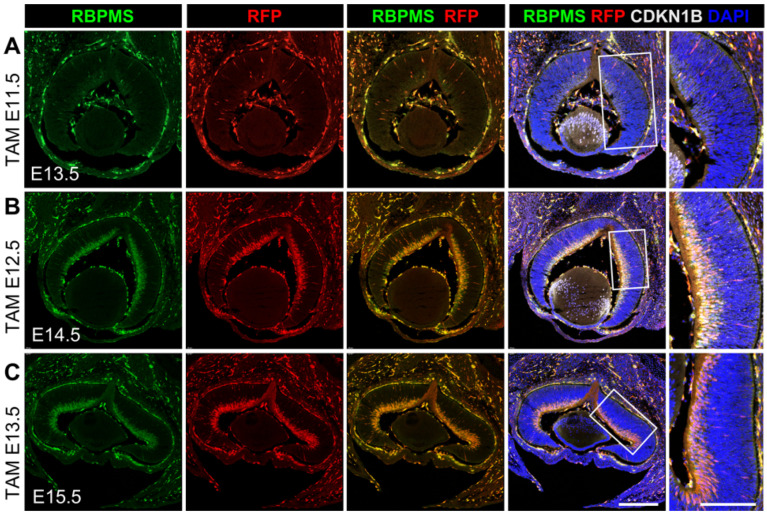
Cre recombinase activity in differentiated RGCs of embryonic *Rbpms^CreERT2/+^*; *Rosa26^td^*^T/+^ mice. Representative images of retinal cryosections of *Rbpms^CreERT2/+^*; *Rosa26^td^*^T/+^ mice immunolabeled with anti-RBPMS (green), anti-RFP (red), and anti-CDKN1B (grey) and nuclear counterstained with DAPI (blue). Tamoxifen was administered at (**A**) E11.5, (**B**) E12.5, and (**C**) E13.5 and retinas were collected for immunolabeling 2 days after tamoxifen induction at E13.5, E14.5, and E15.5, respectively. Far right panels show the enlarged views of the boxed regions. *n* = 3. Scale bars equal 250 μm.

During retinogenesis, RGCs are specified from multipotent retinal progenitor cells (RPCs) in the neuroblast layer, and the newly generated RGCs migrate and settle in the inner layer that becomes the future GCL. RPCs go through a process called interkinetic nuclear migration (INM), during which the RPCs divide and oscillate their nuclei across the retina throughout the cell cycle [28]. In our study, we observed that the RBPMS-driven tdTomato signal was detected in the neuronal blast layer (NBL) and the presumable GCL in central retina at the onset of RGC genesis at E11.5 and propagated towards the peripheral retina (Figure 6B–D). To determine whether *Rbpms*-CreER^T2^ activate tdTomato expression in RPCs, we treated pregnant *Rbpms^CreERT2/+^*; *Rosa26^tdT/+^* mice with a single dosage of tamoxifen and collected retinas two days after each injection for co-immunolabeling with antibodies against RBPMS, RFP, and the postmitotic cell cycle marker CDKN1B (also known as p27Kip1). As shown in Figure 7, in the retinas treated with a single dose of tamoxifen at E11.5, E12.5, and E13.5, nearly all RBPMS^+^ cells in the NBL and in the presumable GCL expressed tdTomato, and the expression of CDKN1B was limited to these RBPMS^+^/tdTomato^+^ cells. These results imply that RBPMS expression starts in the nascent RGCs newly differentiated from the postmitotic RPCs in the NBL and is maintained in the RGCs as they migrate to the GCL. Overall, the inducible *Rbpms*-CreER^T2^ mice display a very high efficiency of Cre recombinase-mediated recombination in both developing and adult retinas upon tamoxifen induction, and the recombination specificity is restricted to RGCs, which make this inducible *Rbpms*-CreER^T2^ mouse line a powerful tool to manipulate genes genetically in retinal disease research.

## 4. Discussion

RBPMS is a common marker for all RGCs, making *Rbpms* locus a strong candidate for generating genetic tool. In the present study, we generated and characterized a novel, RGC-specific inducible Cre mouse line that exhibits a high Cre recombinase activity in all RGCs. We have shown that both heterozygous *Rbpms^CreERT2/+^* and homozygous *Rbpms^CreERT2/CreERT2^* knock-in mice are viable and fertile with no detectable phenotype in retinal morphology, visual function, and transcriptomic profiles, thus making this *Rbpms*-CreER^T2^ mouse line ideal for effective gene manipulation in all RGCs.

Previous work established that *Rbpms* mediates synapse density and axon arbor formation in RGCs and depletion of *Rbpms* leads to an increased density of presynaptic puncta and an enhanced early visual behavior confirmed via OMR assay in zebrafish embryos [70]. In addition, *Rbpms* is implicated in the development of oocyte, heart, and other smooth muscle-rich tissues in several vertebrate species [46,71,72,73], and the deletion of *Rbpms* leads to perinatal lethality in mice resulting from congenital cardiovascular defects [47]. Therefore, we have chosen to knock in the P2A-CreER^T2^ sequence in in-frame fusion to the C-terminus of RBPMS, which does not disrupt *Rbpms* and permits the co-expression of RBPMS and CreER^T2^ through P2A self-cleaving peptide. As expected, both heterozygous *Rbpms^CreERT2/+^* and homozygous *Rbpms^CreERT2/CreERT2^* knock-in mice are viable and devoid of the lethal phenotype associated with the *Rbpms* knockout mutation. Our histological analysis of H&E staining and OCT has further shown that P2A-CreER^T2^ knock-in does not affect the thickness and the number of cells in each retinal layer, including those of ONL, INL, and GCL, nor does it alter visual function as assessed by OMR and ERG.

In our study, we did not detect a change in the cell number of RBPMS^+^ RGCs, nor did we observe a difference in immunolabeling signal intensity of RBPMS in RGCs among wild type, heterozygous *Rbpms^CreERT2/+^*, and homozygous *Rbpms^CreERT2/CreERT2^* knock-in mice. We have further profiled the transcriptomes of these retinas and have compared the expression of genes associated with each major retinal cell type, including genes expressed in all or subtypes of RGCs, such as *Thy1*, *Tubb*3, *Pou4f1-f3*, *Sox4*, *Sox11-12*, *Isl1-2*, *Cartpt*, *Col25a1*, *Fstl4*, *Npy*, *Mmp17*, *Jam2*, *Pvalb*, *Barhl2*, and *Pde1a*. Compared to the controls, the expression level of these genes is not significantly changed in heterozygous *Rbpms^CreERT2/+^* and homozygous *Rbpms^CreERT2/CreERT2^* knock-in mice, further arguing that using P2A in *Rbpms*-CreER^T2^ knock-in results in the co-expression of RBPMS and CreER^T2^ and thus preserves the expression and function of *Rbpms*. Nevertheless, RGCs represent only a small fraction of retinal cells, it is possible that potential changes in the expression of some genes in RGCs can be missed if these genes are expressed in other retinal cell types and their expression do not change in other cell types. Future RNA-Seq analysis using purified RGCs will provide a more accurate detection of possible changes in the transcriptome of *Rbpms*-CreER^T2^ knock-in RGCs.

Among the existing Cre lines with Cre activities in RGCs driven by other gene promotors, *Chx10*/*Vsx2* is expressed in early progenitor cells in retinal development and thus drives Cre expression in most retinal cells born from *Vsx2*^+^ progenitors [74]. *Math5*-Cre labels RGCs as well as cone, horizontal cells, and amacrine cells [75,76]. Similar to *Chx10*/*Vsx2*, *Pax6* drives the expression of Cre in retinal progenitors and group of postmitotic RGC precursors from E9.5 [43], and *Chrnb3*-Cre [44], *Six3*-Cre [77], and *vGluT3*-Cre [78] lines are either expressed in multiple retinal cell types or in selected RGC subtypes. Therefore, due to their broad expression pattern in the retina or limited expression in RGC subsets, the above Cre lines are not suitable to manipulate genes spatiotemporally in RGC development and physiological disease modeling. In contrast, *Rbpms*-CreER^T2^ expression is restricted to RGCs and not in any other retinal cells throughout developing and adult retinas. In our present study, we have assessed the induction of *Rbpms*-CreER^T2^ recombinase activity in adult and embryonic RGCs with different tamoxifen dosages, demonstrating that a single dosage of tamoxifen would be sufficient for gene manipulation in most RGCs and making the *Rbpms*-CreER^T2^ an excellent tool to study gene function in RGCs.

Though this study largely focuses on retinas, the expression of *Rbpms* is also detected in SMCs. Similarly to our recently generated SMC-specific *Itga8*-CreER^T2^ mouse line [79], this *Rbpms*-CreER^T2^ line could be a powerful tool in the research field of SMCs as well. However, the full characterization of *Rbpms*-CreER^T2^ knock-in mice in SMCs and of the recombination efficiency in SMCs remains to be completed.

## 5. Conclusions

In summary, we generated and characterized a novel, RGC-specific inducible Cre mouse line by in-frame knock-in of CreER^T2^ sequence at the C-terminus of RBPMS, which is identified as a pan-RGC marker. The viable and fertile knock-in mice present no overt phenotypes in basic physiological features, retinal structure, whole retinal transcriptome, and visual function. Importantly, the *Rbpms*-CreER^T2^ recombinase exhibits a high recombination efficiency with the activation of tdTomato reporter expression in nearly all RGCs. These results further support this *Rbpms*-CreER^T2^ mouse line as a powerful tool for studying gene function spatiotemporally in RGCs. 

## Data Availability

The original data for the strand-specific RNA sequencing are available at NCBI Gene Expression Omnibus (GEO) database under accession number GSE232726.

## Supplementary Materials

The following supporting information can be downloaded at: https://www.mdpi.com/article/10.3390/cells12151951/s1, Figure S1: Generation and basic physiology assessment of *Rbpms*-CreER^T2^ mice. Figure S2: Absence of Cre recombinase activity in uninduced *Rbpms*-CreER^T2^/+; Rosa26^tdT/+^ mice. Table S1: Relative cell number. Table S2: Relative amplitude of a wave and b wave in scotopic ffERG. Table S3: Relative amplitude of a wave and b wave in photopic ffERG. Table S4: Relative amplitude of P1-N2 in PERG.
