# Supplementary material for: Inducible Rbpms-CreERT2 Mouse Line for Studying Gene Function in Retinal Ganglion Cell Physiology and Disease
Source: Cells. 2023 Jul 27;12(15):1951. doi: 10.3390/cells12151951 (PMC10416940; doi:10.3390/cells12151951)
Supplement: Supplementary file 1 [file cells-12-01951-s001.zip › Figure S2. Absence of Cre recombinase activity in uninduced Rbpms-CreERT2_+; Rosa26tdT_+ mice. .pdf]

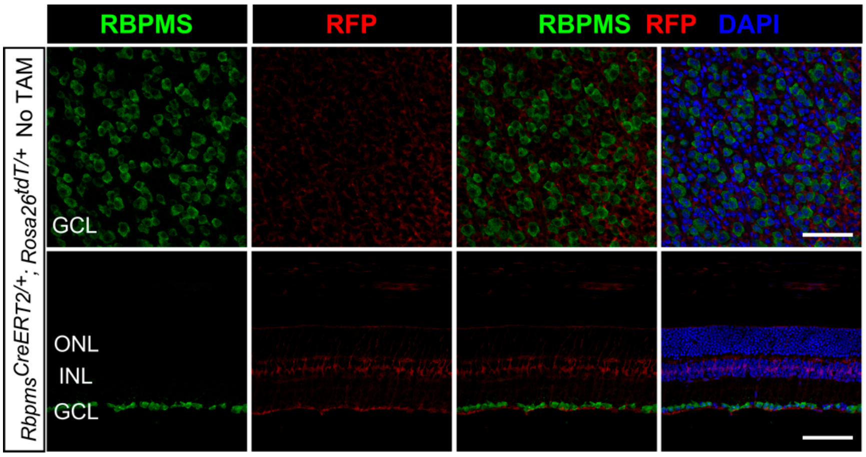

Figure S2. Absence of Cre recombinase activity in uninduced *Rbpms*$^{CreERT2/+}$; *Rosa26*$^{tdT/+}$ mice. Representative images of the ganglion cell layer (GCL) of retinal flat-mounts (top panels) and cryosections (bottom panels) immunolabeled with antibodies against RFP (red) and RBPMS (green) and nuclear counterstained with DAPI (blue). Without tamoxifen, no RFP signal was detected in retinal cells above the background. ONL and INL indicate outer nuclear layer and inner nuclear layer, respectively. Scale bars equal 50 µm.
