# Supplementary material for: Inducible Rbpms-CreERT2 Mouse Line for Studying Gene Function in Retinal Ganglion Cell Physiology and Disease
Source: Cells. 2023 Jul 27;12(15):1951. doi: 10.3390/cells12151951 (PMC10416940; doi:10.3390/cells12151951)
Supplement: Supplementary file 1 [file cells-12-01951-s001.zip › Table S1. Relative cell number.pdf]

Table S1. Relative cell number.

	+/+	*Rbpms*$^{CreERT2/+}$	*Rbpms*$^{CreERT2/CreERT2}$
^{WM}RBPMS^{+}	304.3 ± 59.7	285.9 ± 51.3 $^{\#}$***P* = 0.3799**	281.3 ± 50.5 $^{\#}$***P* = 0.2782**
^{WM}POU4F1^{+}	245.7 ± 55.7	230.7 ± 48.3 $^{\#}$***P* = 0.4711**	237.3 ± 49.0 $^{\#}$***P* = 0.7901**
H&E (in GCL)	45.1 ± 8.3	45.3 ± 7.4 $^{\#}$***P* = 0.9965**	41.4 ± 8.6 $^{\#}$***P* = 0.3138**
H&E (in INL)	546.0 ± 34.8	573.0 ± 12.4 $^{\#}$***P* = 0.7132**	546.3 ± 29.0 $^{\#}$***P* > 0.9999**
H&E (in ONL)	1589.3 ± 62.2	1504.0 ± 16.3 $^{\#}$***P* = 0.1696**	1578.7 ± 50.0 $^{\#}$***P* = 0.9566**

WM indicates whole mount; H&E means hematoxylin and eosin. GCL indicates ganglion cell layer, INL and ONL denote inner nuclear layer and outer nuclear layer, respectively. $^{\#}$ represent *P* value *v.s.* +/+. Data represent the mean ± SEM.
