# Supplementary material for: Inducible Rbpms-CreERT2 Mouse Line for Studying Gene Function in Retinal Ganglion Cell Physiology and Disease
Source: Cells. 2023 Jul 27;12(15):1951. doi: 10.3390/cells12151951 (PMC10416940; doi:10.3390/cells12151951)
Supplement: Supplementary file 1 [file cells-12-01951-s001.zip › Table S2. Relative amplitude of a wave and b wave in scotopic ffERG.pdf]

Table S2. Relative amplitude of a wave and b wave in scotopic ffERG.

Amplitude (μV)	Luminance ($cd.s/m^2$)	+/+	*Rbpms*$^{CreERT2/+}$	*Rbpms*$^{CreERT2/CreERT2}$
a wave (negative)	0.001	52.1 ± 3.3	43.3 ± 8.2 $^{\#}$**P = 0.8951**	41.8 ± 8.1 $^{\#}$**P = 0.8587**
	0.005	19.9 ± 7.8	21.2 ± 7.4 $^{\#}$**P = 0.9978**	21.4 ± 9.2 $^{\#}$**P = 0.9971**
	0.01	42.9 ± 6.6	39.0 ± 7.3 $^{\#}$**P = 0.9778**	55.0 ± 8.6 $^{\#}$**P = 0.8134**
	0.1	231.5 ± 32.4	213.0 ± 29.1 $^{\#}$**P = 0.6118**	225.0 ± 26.2 $^{\#}$**P = 0.9393**
	0.5	344.4 ± 17.9	307.0 ± 27.9 $^{\#}$**P = 0.1469**	321.2 ± 35.7 $^{\#}$**P = 0.4679**
	1	346.9 ± 27.9	321.5 ± 32.1 $^{\#}$**P = 0.4050**	338.0 ± 55.7 $^{\#}$**P = 0.8912**
b wave (positive)	0.001	432.1 ± 49.3	400.5 ± 37.8 $^{\#}$**P = 0.8421**	423.1 ± 67.3 $^{\#}$**P = 0.9862**
	0.005	416.7 ± 54.3	380.0 ± 41.0 $^{\#}$**P = 0.7889**	416.0 ± 49.3 $^{\#}$**P = 0.9999**
	0.01	429.0 ± 65.9	435.0 ± 43.4 $^{\#}$**P = 0.9930**	461.5 ± 47.1 $^{\#}$**P = 0.8295**
	0.1	547.9 ± 128.0	552.3 ± 43.0 $^{\#}$**P = 0.9966**	581.5 ± 79.6 $^{\#}$**P = 0.8233**
	0.5	687.4 ± 63.0	641.1 ± 28.4 $^{\#}$**P = 0.6933**	671.2 ± 87.3 $^{\#}$**P = 0.9561**
	1	682.1 ± 106.4	671.8 ± 25.0 $^{\#}$**P = 0.9816**	679.1 ± 115.8 $^{\#}$**P = 0.9984**

ffERG indicates full-field electroretinography. $^{\#}$ represent *P* value *v.s.* +/+. Data represent the mean ± SEM.
