# Supplementary material for: Inducible Rbpms-CreERT2 Mouse Line for Studying Gene Function in Retinal Ganglion Cell Physiology and Disease
Source: Cells. 2023 Jul 27;12(15):1951. doi: 10.3390/cells12151951 (PMC10416940; doi:10.3390/cells12151951)
Supplement: Supplementary file 1 [file cells-12-01951-s001.zip › Table S3. Relative amplitude of a wave and b wave in photopic ffERG.pdf]

Table S3. Relative amplitude of a wave and b wave in photopic ffERG.

Amplitude (μV)	Luminance (cd.s/m^2)	+/+	*Rbpms*$^{CreERT2/+}$	*Rbpms*$^{CreERT2/CreERT2}$
a wave (negative)	3	15.4 ± 3.6	18.4 ± 2.5 #*P* = 0.6857	14.2 ± 5.7 #*P* = 0.9458
	10	23.0 ± 2.2	22.3 ± 2.5 #*P* = 0.9777	17.9 ± 4.9 #*P* = 0.3396
	25	25.6 ± 2.3	22.8 ± 1.8 #*P* = 0.7276	23.3 ± 5.1 #*P* = 0.8068
	50	28.3 ± 1.1	25.8 ± 3.2 #*P* = 0.7820	28.9 ± 8.4 #*P* = 0.9816
	100	29.7 ± 2.2	29.8 ± 2.5 #*P* = 0.9996	26.5 ± 6.5 #*P* = 0.6493
	150	31.5 ± 1.6	27.0 ± 4.5 #*P* = 0.4285	27.9 ± 8.7 #*P* = 0.5696
b wave (positive)	3	113.8 ± 18.1	107.8 ± 14.9 #*P* = 0.9440	98.4 ± 22.6 #*P* = 0.6826
	10	132.6 ± 19.8	126.5 ± 15.1 #*P* = 0.9421	111.2 ± 26.6 #*P* = 0.4819
	25	154.5 ± 16.6	135.6 ± 19.9 #*P* = 0.5629	121.3 ± 33.2 #*P* = 0.1793
	50	160.4 ± 17.3	141.2 ± 13.0 #*P* = 0.5553	130.6 ± 36.4 #*P* = 0.2483
	100	166.2 ± 16.8	144.3 ± 14.1 #*P* = 0.4668	134.5 ± 31.0 #*P* = 0.2075
	150	176.3 ± 14.0	148.8 ± 17.5 #*P* = 0.3049	137.2 ± 35.6 #*P* = 0.0957

ffERG indicates full-field electroretinography. # represent *P* value *v.s.* +/+. Data represent the mean ± SEM.
