# Supplementary material for: Inducible Rbpms-CreERT2 Mouse Line for Studying Gene Function in Retinal Ganglion Cell Physiology and Disease
Source: Cells. 2023 Jul 27;12(15):1951. doi: 10.3390/cells12151951 (PMC10416940; doi:10.3390/cells12151951)
Supplement: Supplementary file 1 [file cells-12-01951-s001.zip › Table S4. Relative amplitude of P1-N2 in PERG.pdf]

Table S4. Relative amplitude of P1-N2 in PERG.

Amplitude (μV)	Luminance (cd.s/m^2)	+/+	*Rbpms*$^{CreERT2/+}$	*Rbpms*$^{CreERT2/CreERT2}$
P1-N2	50	21.4 ± 5.9	30.9 ± 8.6 $^{\#}$***P* = 0.0917**	27.5 ± 9.0 $^{\#}$***P* = 0.3421**

PERG indicates pattern electroretinography. $^{\#}$ represent *P* value *v.s.* +/+. Data represent the mean ± SEM.

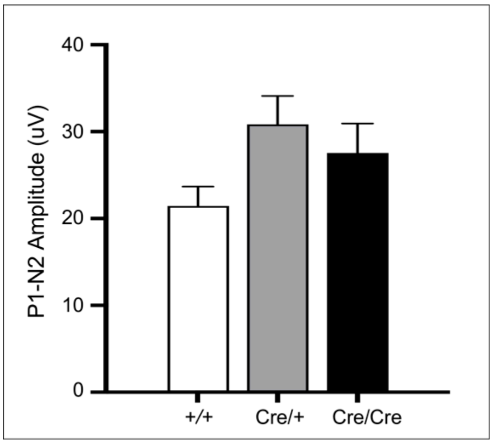

The P1-N2 amplitude was measured from the P1 peak to the nadir of N2.
