# Supplementary material for: Inducible Rbpms-CreERT2 Mouse Line for Studying Gene Function in Retinal Ganglion Cell Physiology and Disease
Source: Cells. 2023 Jul 27;12(15):1951. doi: 10.3390/cells12151951 (PMC10416940; doi:10.3390/cells12151951)
Supplement: Supplementary file 1 [file Figure S1. Generation and basic physiology assessment of Rbpms-CreERT2 mice. .pdf › Figure S1. Generation and basic physiology assessment of Rbpms-CreERT2 mice. .pdf]

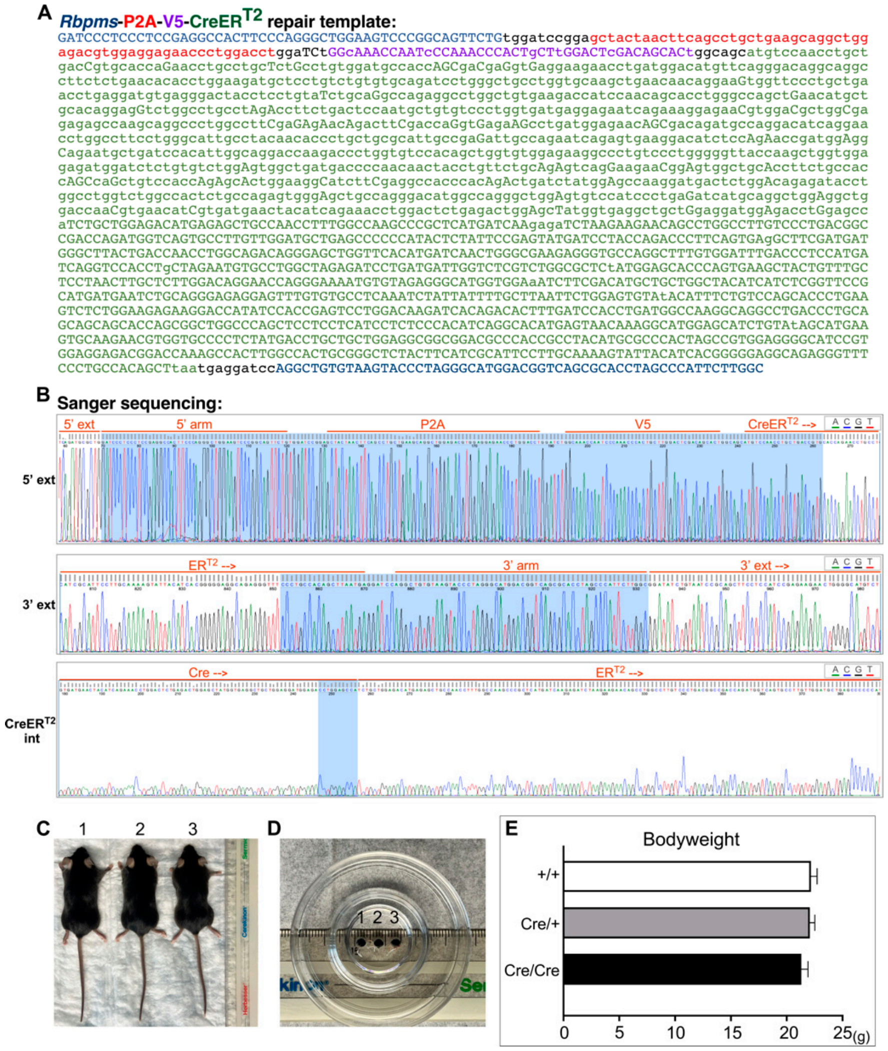

Figure S1. Generation and basic physiology assessment of *Rbpms*-CreER^{T2} mice. (A) Sequence of *Rbpms*-CreER^{T2} knock-in DNA repair template. (B) Sanger sequencing confirms the correctly targeted *Rbpms*-CreER^{T2} knock-in allele. (C-E) Gross morphological analysis. No significant changes in body size (C), eyeball size (D) and the body weight (E) was observed among (1) wild type, (2) *Rbpms*$^{CreERT2/+}$ and (3) *Rbpms*$^{CreERT2/CreERT2}$ mice. $n = 4$. Each bar represents the mean ± SEM. Statistical significance was assessed by one-way ANOVA followed by Tukey's multiple comparisons test.
